# A large-scale comparison of human-written versus ChatGPT-generated essays

**DOI:** 10.1038/s41598-023-45644-9

**Published:** 2023-10-30

**Authors:** Steffen Herbold, Annette Hautli-Janisz, Ute Heuer, Zlata Kikteva, Alexander Trautsch

**Affiliations:** Faculty of Computer Science and Mathematics, University of Passau, Passau, Germany

**Keywords:** Computer science, Information technology

## Abstract

ChatGPT and similar generative AI models have attracted hundreds of millions of users and have become part of the public discourse. Many believe that such models will disrupt society and lead to significant changes in the education system and information generation. So far, this belief is based on either colloquial evidence or benchmarks from the owners of the models—both lack scientific rigor. We systematically assess the quality of AI-generated content through a large-scale study comparing human-written versus ChatGPT-generated argumentative student essays. We use essays that were rated by a large number of human experts (teachers). We augment the analysis by considering a set of linguistic characteristics of the generated essays. Our results demonstrate that ChatGPT generates essays that are rated higher regarding quality than human-written essays. The writing style of the AI models exhibits linguistic characteristics that are different from those of the human-written essays. Since the technology is readily available, we believe that educators must act immediately. We must re-invent homework and develop teaching concepts that utilize these AI models in the same way as math utilizes the calculator: teach the general concepts first and then use AI tools to free up time for other learning objectives.

## Introduction

The massive uptake in the development and deployment of large-scale Natural Language Generation (NLG) systems in recent months has yielded an almost unprecedented worldwide discussion of the future of society. The ChatGPT service which serves as Web front-end to GPT-3.5^1^ and GPT-4 was the fastest-growing service in history to break the 100 million user milestone in January and had 1 billion visits by February 2023^2^.

Driven by the upheaval that is particularly anticipated for education^3^ and knowledge transfer for future generations, we conduct the first independent, systematic study of AI-generated language content that is typically dealt with in high-school education: argumentative essays, i.e. essays in which students discuss a position on a controversial topic by collecting and reflecting on evidence (e.g. ‘Should students be taught to cooperate or compete?’). Learning to write such essays is a crucial aspect of education, as students learn to systematically assess and reflect on a problem from different perspectives. Understanding the capability of generative AI to perform this task increases our understanding of the skills of the models, as well as of the challenges educators face when it comes to teaching this crucial skill. While there is a multitude of individual examples and anecdotal evidence for the quality of AI-generated content in this genre (e.g.^4^) this paper is the first to systematically assess the quality of human-written and AI-generated argumentative texts across different versions of ChatGPT^5^. We use a fine-grained essay quality scoring rubric based on content and language mastery and employ a significant pool of domain experts, i.e. high school teachers across disciplines, to perform the evaluation. Using computational linguistic methods and rigorous statistical analysis, we arrive at several key findings:

- AI models generate significantly higher-quality argumentative essays than the users of an essay-writing online forum frequented by German high-school students across all criteria in our scoring rubric.
- ChatGPT-4 (ChatGPT web interface with the GPT-4 model) significantly outperforms ChatGPT-3 (ChatGPT web interface with the GPT-3.5 default model) with respect to logical structure, language complexity, vocabulary richness and text linking.
- Writing styles between humans and generative AI models differ significantly: for instance, the GPT models use more nominalizations and have higher sentence complexity (signaling more complex, ‘scientific’, language), whereas the students make more use of modal and epistemic constructions (which tend to convey speaker attitude).
- The linguistic diversity of the NLG models seems to be improving over time: while ChatGPT-3 still has a significantly lower linguistic diversity than humans, ChatGPT-4 has a significantly higher diversity than the students.

Our work goes significantly beyond existing benchmarks. While OpenAI’s technical report on GPT-4^6^ presents some benchmarks, their evaluation lacks scientific rigor: it fails to provide vital information like the agreement between raters, does not report on details regarding the criteria for assessment or to what extent and how a statistical analysis was conducted for a larger sample of essays. In contrast, our benchmark provides the first (statistically) rigorous and systematic study of essay quality, paired with a computational linguistic analysis of the language employed by humans and two different versions of ChatGPT, offering a glance at how these NLG models develop over time. While our work is focused on argumentative essays in education, the genre is also relevant beyond education. In general, studying argumentative essays is one important aspect to understand how good generative AI models are at conveying arguments and, consequently, persuasive writing in general.

## Related work

### Natural language generation

The recent interest in generative AI models can be largely attributed to the public release of ChatGPT, a public interface in the form of an interactive chat based on the InstructGPT^1^ model, more commonly referred to as GPT-3.5. In comparison to the original GPT-3^7^ and other similar generative large language models based on the transformer architecture like GPT-J^8^, this model was not trained in a purely self-supervised manner (e.g. through masked language modeling). Instead, a pipeline that involved human-written content was used to fine-tune the model and improve the quality of the outputs to both mitigate biases and safety issues, as well as make the generated text more similar to text written by humans. Such models are referred to as Fine-tuned LAnguage Nets (FLANs). For details on their training, we refer to the literature^9^. Notably, this process was recently reproduced with publicly available models such as Alpaca^10^ and Dolly (i.e. the complete models can be downloaded and not just accessed through an API). However, we can only assume that a similar process was used for the training of GPT-4 since the paper by OpenAI does not include any details on model training.

Testing of the language competency of large-scale NLG systems has only recently started. Cai et al.^11^ show that ChatGPT reuses sentence structure, accesses the intended meaning of an ambiguous word, and identifies the thematic structure of a verb and its arguments, replicating human language use. Mahowald^12^ compares ChatGPT’s acceptability judgments to human judgments on the Article + Adjective + Numeral + Noun construction in English. Dentella et al.^13^ show that ChatGPT-3 fails to understand low-frequent grammatical constructions like complex nested hierarchies and self-embeddings. In another recent line of research, the structure of automatically generated language is evaluated. Guo et al.^14^ show that in question-answer scenarios, ChatGPT-3 uses different linguistic devices than humans. Zhao et al.^15^ show that ChatGPT generates longer and more diverse responses when the user is in an apparently negative emotional state.

Given that we aim to identify certain linguistic characteristics of human-written versus AI-generated content, we also draw on related work in the field of linguistic fingerprinting, which assumes that each human has a unique way of using language to express themselves, i.e. the linguistic means that are employed to communicate thoughts, opinions and ideas differ between humans. That these properties can be identified with computational linguistic means has been showcased across different tasks: the computation of a linguistic fingerprint allows to distinguish authors of literary works^16^, the identification of speaker profiles in large public debates^17–20^ and the provision of data for forensic voice comparison in broadcast debates^21,22^. For educational purposes, linguistic features are used to measure essay readability^23^, essay cohesion^24^ and language performance scores for essay grading^25^. Integrating linguistic fingerprints also yields performance advantages for classification tasks, for instance in predicting user opinion^26,27^ and identifying individual users^28^.

### Limitations of OpenAIs ChatGPT evaluations

OpenAI published a discussion of the model’s performance of several tasks, including Advanced Placement (AP) classes within the US educational system^6^. The subjects used in performance evaluation are diverse and include arts, history, English literature, calculus, statistics, physics, chemistry, economics, and US politics. While the models achieved good or very good marks in most subjects, they did not perform well in English literature. GPT-3.5 also experienced problems with chemistry, macroeconomics, physics, and statistics. While the overall results are impressive, there are several significant issues: firstly, the conflict of interest of the model’s owners poses a problem for the performance interpretation. Secondly, there are issues with the soundness of the assessment beyond the conflict of interest, which make the generalizability of the results hard to assess with respect to the models’ capability to write essays. Notably, the AP exams combine multiple-choice questions with free-text answers. Only the aggregated scores are publicly available. To the best of our knowledge, neither the generated free-text answers, their overall assessment, nor their assessment given specific criteria from the used judgment rubric are published. Thirdly, while the paper states that 1–2 qualified third-party contractors participated in the rating of the free-text answers, it is unclear how often multiple ratings were generated for the same answer and what was the agreement between them. This lack of information hinders a scientifically sound judgement regarding the capabilities of these models in general, but also specifically for essays. Lastly, the owners of the model conducted their study in a few-shot prompt setting, where they gave the models a very structured template as well as an example of a human-written high-quality essay to guide the generation of the answers. This further fine-tuning of what the models generate could have also influenced the output. The results published by the owners go beyond the AP courses which are directly comparable to our work and also consider other student assessments like Graduate Record Examinations (GREs). However, these evaluations suffer from the same problems with the scientific rigor as the AP classes.

### Scientific assessment of ChatGPT

Researchers across the globe are currently assessing the individual capabilities of these models with greater scientific rigor. We note that due to the recency and speed of these developments, the hereafter discussed literature has mostly only been published as pre-prints and has not yet been peer-reviewed. In addition to the above issues concretely related to the assessment of the capabilities to generate student essays, it is also worth noting that there are likely large problems with the trustworthiness of evaluations, because of data contamination, i.e. because the benchmark tasks are part of the training of the model, which enables memorization. For example, Aiyappa et al.^29^ find evidence that this is likely the case for benchmark results regarding NLP tasks. This complicates the effort by researchers to assess the capabilities of the models beyond memorization.

Nevertheless, the first assessment results are already available – though mostly focused on ChatGPT-3 and not yet ChatGPT-4. Closest to our work is a study by Yeadon et al.^30^, who also investigate ChatGPT-3 performance when writing essays. They grade essays generated by ChatGPT-3 for five physics questions based on criteria that cover academic content, appreciation of the underlying physics, grasp of subject material, addressing the topic, and writing style. For each question, ten essays were generated and rated independently by five researchers. While the sample size precludes a statistical assessment, the results demonstrate that the AI model is capable of writing high-quality physics essays, but that the quality varies in a manner similar to human-written essays.

Guo et al.^14^ create a set of free-text question answering tasks based on data they collected from the internet, e.g. question answering from Reddit. The authors then sample thirty triplets of a question, a human answer, and a ChatGPT-3 generated answer and ask human raters to assess if they can detect which was written by a human, and which was written by an AI. While this approach does not directly assess the quality of the output, it serves as a Turing test^31^ designed to evaluate whether humans can distinguish between human- and AI-produced output. The results indicate that humans are in fact able to distinguish between the outputs when presented with a pair of answers. Humans familiar with ChatGPT are also able to identify over 80% of AI-generated answers without seeing a human answer in comparison. However, humans who are not yet familiar with ChatGPT-3 are not capable of identifying AI-written answers about 50% of the time. Moreover, the authors also find that the AI-generated outputs are deemed to be more helpful than the human answers in slightly more than half of the cases. This suggests that the strong results from OpenAI’s own benchmarks regarding the capabilities to generate free-text answers generalize beyond the benchmarks.

There are, however, some indicators that the benchmarks may be overly optimistic in their assessment of the model’s capabilities. For example, Kortemeyer^32^ conducts a case study to assess how well ChatGPT-3 would perform in a physics class, simulating the tasks that students need to complete as part of the course: answer multiple-choice questions, do homework assignments, ask questions during a lesson, complete programming exercises, and write exams with free-text questions. Notably, ChatGPT-3 was allowed to interact with the instructor for many of the tasks, allowing for multiple attempts as well as feedback on preliminary solutions. The experiment shows that ChatGPT-3’s performance is in many aspects similar to that of the beginning learners and that the model makes similar mistakes, such as omitting units or simply plugging in results from equations. Overall, the AI would have passed the course with a low score of 1.5 out of 4.0. Similarly, Kung et al.^33^ study the performance of ChatGPT-3 in the United States Medical Licensing Exam (USMLE) and find that the model performs at or near the passing threshold. Their assessment is a bit more optimistic than Kortemeyer’s as they state that this level of performance, comprehensible reasoning and valid clinical insights suggest that models such as ChatGPT may potentially assist human learning in clinical decision making.

Frieder et al.^34^ evaluate the capabilities of ChatGPT-3 in solving graduate-level mathematical tasks. They find that while ChatGPT-3 seems to have some mathematical understanding, its level is well below that of an average student and in most cases is not sufficient to pass exams. Yuan et al.^35^ consider the arithmetic abilities of language models, including ChatGPT-3 and ChatGPT-4. They find that they exhibit the best performance among other currently available language models (incl. Llama^36^, FLAN-T5^37^, and Bloom^38^). However, the accuracy of basic arithmetic tasks is still only at 83% when considering correctness to the degree of $10^{-3}$, i.e. such models are still not capable of functioning reliably as calculators. In a slightly satiric, yet insightful take, Spencer et al.^39^ assess how a scientific paper on gamma-ray astrophysics would look like, if it were written largely with the assistance of ChatGPT-3. They find that while the language capabilities are good and the model is capable of generating equations, the arguments are often flawed and the references to scientific literature are full of hallucinations.

The general reasoning skills of the models may also not be at the level expected from the benchmarks. For example, Cherian et al.^40^ evaluate how well ChatGPT-3 performs on eleven puzzles that second graders should be able to solve and find that ChatGPT is only able to solve them on average in 36.4% of attempts, whereas the second graders achieve a mean of 60.4%. However, their sample size is very small and the problem was posed as a multiple-choice question answering problem, which cannot be directly compared to the NLG we consider.

### Research gap

Within this article, we address an important part of the current research gap regarding the capabilities of ChatGPT (and similar technologies), guided by the following research questions:

- **RQ1:** How good is ChatGPT based on GPT-3 and GPT-4 at writing argumentative student essays?
- **RQ2:** How do AI-generated essays compare to essays written by students?
- **RQ3:** What are linguistic devices that are characteristic of student versus AI-generated content?

We study these aspects with the help of a large group of teaching professionals who systematically assess a large corpus of student essays. To the best of our knowledge, this is the first large-scale, independent scientific assessment of ChatGPT (or similar models) of this kind. Answering these questions is crucial to understanding the impact of ChatGPT on the future of education.

## Materials and methods

### Data

The essay topics originate from a corpus of argumentative essays in the field of argument mining^41^. Argumentative essays require students to think critically about a topic and use evidence to establish a position on the topic in a concise manner. The corpus features essays for 90 topics from Essay Forum^42^, an active community for providing writing feedback on different kinds of text and is frequented by high-school students to get feedback from native speakers on their essay-writing capabilities. Information about the age of the writers is not available, but the topics indicate that the essays were written in grades 11–13, indicating that the authors were likely at least 16. Topics range from ‘Should students be taught to cooperate or to compete?’ to ‘Will newspapers become a thing of the past?’. In the corpus, each topic features one human-written essay uploaded and discussed in the forum. The students who wrote the essays are not native speakers. The average length of these essays is 19 sentences with 388 tokens (an average of 2.089 characters) and will be termed ‘student essays’ in the remainder of the paper.

For the present study, we use the topics from Stab and Gurevych^41^ and prompt ChatGPT with ‘Write an essay with about 200 words on “[topic]”’ to receive automatically-generated essays from the ChatGPT-3 and ChatGPT-4 versions from 22 March 2023 (‘ChatGPT-3 essays’, ‘ChatGPT-4 essays’). No additional prompts for getting the responses were used, i.e. the data was created with a basic prompt in a zero-shot scenario. This is in contrast to the benchmarks by OpenAI, who used an engineered prompt in a few-shot scenario to guide the generation of essays. We note that we decided to ask for 200 words because we noticed a tendency to generate essays that are longer than the desired length by ChatGPT. A prompt asking for 300 words typically yielded essays with more than 400 words. Thus, using the shorter length of 200, we prevent a potential advantage for ChatGPT through longer essays, and instead err on the side of brevity. Similar to the evaluations of free-text answers by OpenAI, we did not consider multiple configurations of the model due to the effort required to obtain human judgments. For the same reason, our data is restricted to ChatGPT and does not include other models available at that time, e.g. Alpaca. We use the browser versions of the tools because we consider this to be a more realistic scenario than using the API. Table 1 below shows the core statistics of the resulting dataset. Supplemental material S1 shows examples for essays from the data set.

**Table 1:** Core statistics of the dataset.

**Source**	**Length (words/essay)**	**Sentences/essay**	**Words/sentence**
Student	339.13	18.98	18.60
ChatGPT-3	247.96	12.40	20.31
ChatGPT-4	253.70	13.08	19.57

### Annotation study

#### Study participants

The participants had registered for a two-hour online training entitled ‘ChatGPT – Challenges and Opportunities’ conducted by the authors of this paper as a means to provide teachers with some of the technological background of NLG systems in general and ChatGPT in particular. Only teachers permanently employed at secondary schools were allowed to register for this training. Focusing on these experts alone allows us to receive meaningful results as those participants have a wide range of experience in assessing students’ writing. A total of 139 teachers registered for the training, 129 of them teach at grammar schools, and only 10 teachers hold a position at other secondary schools. About half of the registered teachers (68 teachers) have been in service for many years and have successfully applied for promotion. For data protection reasons, we do not know the subject combinations of the registered teachers. We only know that a variety of subjects are represented, including languages (English, French and German), religion/ethics, and science. Supplemental material S5 provides some general information regarding German teacher qualifications.

The training began with an online lecture followed by a discussion phase. Teachers were given an overview of language models and basic information on how ChatGPT was developed. After about 45 minutes, the teachers received a both written and oral explanation of the questionnaire at the core of our study (see Supplementary material S3) and were informed that they had 30 minutes to finish the study tasks. The explanation included information on how the data was obtained, why we collect the self-assessment, and how we chose the criteria for the rating of the essays, the overall goal of our research, and a walk-through of the questionnaire. Participation in the questionnaire was voluntary and did not affect the awarding of a training certificate. We further informed participants that all data was collected anonymously and that we would have no way of identifying who participated in the questionnaire. We orally informed participants that they consent to the use of the provided ratings for our research by participating in the survey.

Once these instructions were provided orally and in writing, the link to the online form was given to the participants. The online form was running on a local server that did not log any information that could identify the participants (e.g. IP address) to ensure anonymity. As per instructions, consent for participation was given by using the online form. Due to the full anonymity, we could by definition not document who exactly provided the consent. This was implemented as further insurance that non-participation could not possibly affect being awarded the training certificate.

About 20% of the training participants did not take part in the questionnaire study, the remaining participants consented based on the information provided and participated in the rating of essays. After the questionnaire, we continued with an online lecture on the opportunities of using ChatGPT for teaching as well as AI beyond chatbots. The study protocol was reviewed and approved by the Research Ethics Committee of the University of Passau. We further confirm that our study protocol is in accordance with all relevant guidelines.

#### Questionnaire

The questionnaire consists of three parts: first, a brief self-assessment regarding the English skills of the participants which is based on the Common European Framework of Reference for Languages (CEFR)^43^. We have six levels ranging from ‘comparable to a native speaker’ to ‘some basic skills’ (see supplementary material S3). Then each participant was shown six essays. The participants were only shown the generated text and were not provided with information on whether the text was human-written or AI-generated.

The questionnaire covers the seven categories relevant for essay assessment shown below (for details see supplementary material S3):

- Topic and completeness
- Logic and composition
- Expressiveness and comprehensiveness
- Language mastery
- Complexity
- Vocabulary and text linking
- Language constructs

These categories are used as guidelines for essay assessment^44^ established by the Ministry for Education of Lower Saxony, Germany. For each criterion, a seven-point Likert scale with scores from zero to six is defined, where zero is the worst score (e.g. no relation to the topic) and six is the best score (e.g. addressed the topic to a special degree). The questionnaire included a written description as guidance for the scoring.

After rating each essay, the participants were also asked to self-assess their confidence in the ratings. We used a five-point Likert scale based on the criteria for the self-assessment of peer-review scores from the Association for Computational Linguistics (ACL). Once a participant finished rating the six essays, they were shown a summary of their ratings, as well as the individual ratings for each of their essays and the information on how the essay was generated.

### Computational linguistic analysis

In order to further explore and compare the quality of the essays written by students and ChatGPT, we consider the six following linguistic characteristics: lexical diversity, sentence complexity, nominalization, presence of modals, epistemic and discourse markers. Those are motivated by previous work: Weiss et al.^25^ observe the correlation between measures of lexical, syntactic and discourse complexities to the essay gradings of German high-school examinations while McNamara et al.^45^ explore cohesion (indicated, among other things, by connectives), syntactic complexity and lexical diversity in relation to the essay scoring.

#### Lexical diversity

We identify vocabulary richness by using a well-established measure of textual, lexical diversity (MTLD)^46^ which is often used in the field of automated essay grading^25,45,47^. It takes into account the number of unique words but unlike the best-known measure of lexical diversity, the type-token ratio (TTR), it is not as sensitive to the difference in the length of the texts. In fact, Koizumi and In’nami^48^ find it to be least affected by the differences in the length of the texts compared to some other measures of lexical diversity. This is relevant to us due to the difference in average length between the human-written and ChatGPT-generated essays.

#### Syntactic complexity

We use two measures in order to evaluate the syntactic complexity of the essays. One is based on the maximum depth of the sentence dependency tree which is produced using the spaCy 3.4.2 dependency parser^49^ (‘Syntactic complexity (depth)’). For the second measure, we adopt an approach similar in nature to the one by Weiss et al.^25^ who use clause structure to evaluate syntactic complexity. In our case, we count the number of conjuncts, clausal modifiers of nouns, adverbial clause modifiers, clausal complements, clausal subjects, and parataxes (‘Syntactic complexity (clauses)’). The supplementary material in S2 shows the difference between sentence complexity based on two examples from the data.

Nominalization is a common feature of a more scientific style of writing^50^ and is used as an additional measure for syntactic complexity. In order to explore this feature, we count occurrences of nouns with suffixes such as ‘-ion’, ‘-ment’, ‘-ance’ and a few others which are known to transform verbs into nouns.

#### Semantic properties

Both modals and epistemic markers signal the commitment of the writer to their statement. We identify modals using the POS-tagging module provided by spaCy as well as a list of epistemic expressions of modality, such as ‘definitely’ and ‘potentially’, also used in other approaches to identifying semantic properties^51^. For epistemic markers we adopt an empirically-driven approach and utilize the epistemic markers identified in a corpus of dialogical argumentation by Hautli-Janisz et al.^52^. We consider expressions such as ‘I think’, ‘it is believed’ and ‘in my opinion’ to be epistemic.

#### Discourse properties

Discourse markers can be used to measure the coherence quality of a text. This has been explored by Somasundaran et al.^53^ who use discourse markers to evaluate the story-telling aspect of student writing while Nadeem et al.^54^ incorporated them in their deep learning-based approach to automated essay scoring. In the present paper, we employ the PDTB list of discourse markers^55^ which we adjust to exclude words that are often used for purposes other than indicating discourse relations, such as ‘like’, ‘for’, ‘in’ etc.

### Statistical methods

We use a within-subjects design for our study. Each participant was shown six randomly selected essays. Results were submitted to the survey system after each essay was completed, in case participants ran out of time and did not finish scoring all six essays. Cronbach’s $\alpha$^56^ allows us to determine the inter-rater reliability for the rating criterion and data source (human, ChatGPT-3, ChatGPT-4) in order to understand the reliability of our data not only overall, but also for each data source and rating criterion. We use two-sided Wilcoxon-rank-sum tests^57^ to confirm the significance of the differences between the data sources for each criterion. We use the same tests to determine the significance of the linguistic characteristics. This results in three comparisons (human vs. ChatGPT-3, human vs. ChatGPT-4, ChatGPT-3 vs. ChatGPT-4) for each of the seven rating criteria and each of the seven linguistic characteristics, i.e. 42 tests. We use the Holm-Bonferroni method^58^ for the correction for multiple tests to achieve a family-wise error rate of 0.05. We report the effect size using Cohen’s *d*^59^. While our data is not perfectly normal, it also does not have severe outliers, so we prefer the clear interpretation of Cohen’s *d* over the slightly more appropriate, but less accessible non-parametric effect size measures. We report point plots with estimates of the mean scores for each data source and criterion, incl. the 95% confidence interval of these mean values. The confidence intervals are estimated in a non-parametric manner based on bootstrap sampling. We further visualize the distribution for each criterion using violin plots to provide a visual indicator of the spread of the data (see Supplementary material S4).

Further, we use the self-assessment of the English skills and confidence in the essay ratings as confounding variables. Through this, we determine if ratings are affected by the language skills or confidence, instead of the actual quality of the essays. We control for the impact of these by measuring Pearson’s correlation coefficient *r*^60^ between the self-assessments and the ratings. We also determine whether the linguistic features are correlated with the ratings as expected. The sentence complexity (both tree depth and dependency clauses), as well as the nominalization, are indicators of the complexity of the language. Similarly, the use of discourse markers should signal a proper logical structure. Finally, a large lexical diversity should be correlated with the ratings for the vocabulary. Same as above, we measure Pearson’s *r*. We use a two-sided test for the significance based on a $\beta$-distribution that models the expected correlations as implemented by scipy^61^. Same as above, we use the Holm-Bonferroni method to account for multiple tests. However, we note that it is likely that all—even tiny—correlations are significant given our amount of data. Consequently, our interpretation of these results focuses on the strength of the correlations.

Our statistical analysis of the data is implemented in Python. We use pandas 1.5.3 and numpy 1.24.2 for the processing of data, pingouin 0.5.3 for the calculation of Cronbach’s $\alpha$, scipy 1.10.1 for the Wilcoxon-rank-sum tests Pearson’s *r*, and seaborn 0.12.2 for the generation of plots, incl. the calculation of error bars that visualize the confidence intervals.

## Results

**Table 2:** Arithmetic mean (M), standard deviation (SD), and Cronbach’s $\alpha$ for the ratings.

**Criterion**	**Humans**			**ChatGPT-3**			**ChatGPT-4**		
	M	SD	$\alpha$	M	SD	$\alpha$	M	SD	$\alpha$
Topic and completeness	3.58	1.30	0.95	4.24	1.16	0.95	4.54	1.12	0.95
Logic and composition	3.64	1.27	0.96	4.29	1.04	0.96	4.64	1.01	0.96
Expressiveness and compr.	3.42	1.25	0.95	3.90	1.04	0.95	4.23	1.12	0.95
Language mastery	3.90	1.37	0.89	5.03	1.19	0.89	5.25	1.08	0.89
Complexity	3.72	1.26	0.92	4.20	1.14	0.92	4.60	1.10	0.92
Vocabulary and text linking	3.78	1.18	0.97	4.41	1.05	0.97	4.81	1.06	0.97
Language constructs	3.80	1.15	0.97	4.47	1.02	0.97	4.73	1.07	0.97
Overall	3.69	1.26		4.36	1.14		4.68	1.11

**Table 3:** Arithmetic mean (M) and standard deviation (SD) for the linguistic markers.

**Linguistic characteristic**	**Humans**		**ChatGPT-3**		**ChatGPT-4**	
Lexical diversity	95.72	23.50	75.68	12.89	108.91	20.73
Syntactic complexity (depth)	5.72	0.80	6.18	0.76	5.94	0.54
Syntactic complexity (clauses)	1.81	0.57	2.31	0.50	2.08	0.42
Nominalizations	1.06	0.51	1.56	0.63	1.73	0.49
Modals	10.84	5.30	8.97	4.21	6.12	3.18
Epistemic markers	0.06	0.06	0.02	0.03	0.00	0.00
Discourse markers	0.57	0.24	0.52	0.19	0.36	0.17

**Table 4:** P-values of the Wilcoxon signed-rank tests adjusted for multiple comparisons using the Holm-Bonferroni method. Effect sizes measured with Cohen’s *d* reported for significant results.

**Criterion/Linguistic characteristic**	**Human vs. ChatGPT-3**	**ChatGPT-3 vs. ChatGPT-4**	**ChatGPT-3 vs. ChatGPT-4**
Topic and completeness	$<0.001 (d=-\,0.77)$	$<0.001 (d=-\,1.09)$	0.095
Logic and composition	$<0.001 (d=-\,0.84)$	$<0.001 (d=-\,1.20)$	$0.025 (d=-\,0.45)$
Expressiveness and compr.	$0.008 (d=-\,0.57)$	$<0.001 (d=-\,0.88)$	0.055
Language mastery	$<0.001 (d=-\,1.15)$	$<0.001 (d=-\,1.43)$	0.105
Complexity	$0.025 (d=-\,0.52)$	$<0.001 (d=-\,0.99)$	$0.025 (d=-\,0.48)$
Vocabulary and text linking	$<0.001 (d=-\,0.76)$	$<0.001 (d=-\,1.27)$	$0.012 (d=-\,0.50)$
Language constructs	$<0.001 (d=-\,0.82)$	$<0.001 (d=-\,1.15)$	0.105
Lexical diversity	$<0.001 (d=1.06)$	$0.001 (d=-\,0.60)$	$<0.001 (d=-\,1.93)$
Syntactic complexity (depth)	$0.001 (d=-\,0.59)$	0.055	0.105
Syntactic complexity (clauses)	$<0.001 (d=-\,0.93)$	$0.004 (d=-\,0.54)$	$0.024 (d=0.49)$
Nominalizations	$<0.001 (d=-\,0.88)$	$<0.001 (d=-\,1.35)$	$0.020 (d=-\,0.29)$
Modals	$0.025 (d=0.39)$	$<0.001 (d=1.08)$	$<0.001 (d=0.76)$
Epistemic markers	$<0.001 (d=1.01)$	$<0.001 (d=1.53)$	$0.005 (d=0.65)$
Discourse markers	0.150	$<0.001 (d=0.98)$	$<0.001 (d=0.85)$

Out of the 111 teachers who completed the questionnaire, 108 rated all six essays, one rated five essays, one rated two essays, and one rated only one essay. This results in 658 ratings for 270 essays (90 topics for each essay type: human-, ChatGPT-3-, ChatGPT-4-generated), with three ratings for 121 essays, two ratings for 144 essays, and one rating for five essays. The inter-rater agreement is consistently excellent ($\alpha >0.9$), with the exception of language mastery where we have good agreement ($\alpha =0.89$, see Table 2). Further, the correlation analysis depicted in supplementary material S4 shows weak positive correlations ($r \in 0.11, 0.28]$) between the self-assessment for the English skills, respectively the self-assessment for the confidence in ratings and the actual ratings. Overall, this indicates that our ratings are reliable estimates of the actual quality of the essays with a potential small tendency that confidence in ratings and language skills yields better ratings, independent of the data source.

Table 2 and supplementary material S4 characterize the distribution of the ratings for the essays, grouped by the data source. We observe that for all criteria, we have a clear order of the mean values, with students having the worst ratings, ChatGPT-3 in the middle rank, and ChatGPT-4 with the best performance. We further observe that the standard deviations are fairly consistent and slightly larger than one, i.e. the spread is similar for all ratings and essays. This is further supported by the visual analysis of the violin plots.

The statistical analysis of the ratings reported in Table 4 shows that differences between the human-written essays and the ones generated by both ChatGPT models are significant. The effect sizes for human versus ChatGPT-3 essays are between 0.52 and 1.15, i.e. a medium ($d \in [0.5,0.8)$) to large ($d \in [0.8, 1.2)$) effect. On the one hand, the smallest effects are observed for the expressiveness and complexity, i.e. when it comes to the overall comprehensiveness and complexity of the sentence structures, the differences between the humans and the ChatGPT-3 model are smallest. On the other hand, the difference in language mastery is larger than all other differences, which indicates that humans are more prone to making mistakes when writing than the NLG models. The magnitude of differences between humans and ChatGPT-4 is larger with effect sizes between 0.88 and 1.43, i.e., a large to very large ($d \in [1.2, 2)$) effect. Same as for ChatGPT-3, the differences are smallest for expressiveness and complexity and largest for language mastery. Please note that the difference in language mastery between humans and both GPT models does not mean that the humans have low scores for language mastery (M=3.90), but rather that the NLG models have exceptionally high scores (M=5.03 for ChatGPT-3, M=5.25 for ChatGPT-4).

When we consider the differences between the two GPT models, we observe that while ChatGPT-4 has consistently higher mean values for all criteria, only the differences for logic and composition, vocabulary and text linking, and complexity are significant. The effect sizes are between 0.45 and 0.5, i.e. small ($d \in [0.2, 0.5)$) and medium. Thus, while GPT-4 seems to be an improvement over GPT-3.5 in general, the only clear indicator of this is a better and clearer logical composition and more complex writing with a more diverse vocabulary.

We also observe significant differences in the distribution of linguistic characteristics between all three groups (see Table 3). Sentence complexity (depth) is the only category without a significant difference between humans and ChatGPT-3, as well as ChatGPT-3 and ChatGPT-4. There is also no significant difference in the category of discourse markers between humans and ChatGPT-3. The magnitude of the effects varies a lot and is between 0.39 and 1.93, i.e., between small ($d \in [0.2, 0.5)$) and very large. However, in comparison to the ratings, there is no clear tendency regarding the direction of the differences. For instance, while the ChatGPT models write more complex sentences and use more nominalizations, humans tend to use more modals and epistemic markers instead. The lexical diversity of humans is higher than that of ChatGPT-3 but lower than that of ChatGPT-4. While there is no difference in the use of discourse markers between humans and ChatGPT-3, ChatGPT-4 uses significantly fewer discourse markers.

We detect the expected positive correlations between the complexity ratings and the linguistic markers for sentence complexity ($r=0.16$ for depth, $r=0.19$ for clauses) and nominalizations ($r=0.22$). However, we observe a negative correlation between the logic ratings and the discourse markers ($r=-0.14$), which counters our intuition that more frequent use of discourse indicators makes a text more logically coherent. However, this is in line with previous work: McNamara et al.^45^ also find no indication that the use of cohesion indices such as discourse connectives correlates with high- and low-proficiency essays. Finally, we observe the expected positive correlation between the ratings for the vocabulary and the lexical diversity ($r=0.12$). All observed correlations are significant. However, we note that the strength of all these correlations is weak and that the significance itself should not be over-interpreted due to the large sample size.

## Discussion

Our results provide clear answers to the first two research questions that consider the quality of the generated essays: ChatGPT performs well at writing argumentative student essays and outperforms the quality of the human-written essays significantly. The ChatGPT-4 model has (at least) a large effect and is on average about one point better than humans on a seven-point Likert scale.

Regarding the third research question, we find that there are significant linguistic differences between humans and AI-generated content. The AI-generated essays are highly structured, which for instance is reflected by the identical beginnings of the concluding sections of all ChatGPT essays (‘In conclusion, [...]’). The initial sentences of each essay are also very similar starting with a general statement using the main concepts of the essay topics. Although this corresponds to the general structure that is sought after for argumentative essays, it is striking to see that the ChatGPT models are so rigid in realizing this, whereas the human-written essays are looser in representing the guideline on the linguistic surface. Moreover, the linguistic fingerprint has the counter-intuitive property that the use of discourse markers is negatively correlated with logical coherence. We believe that this might be due to the rigid structure of the generated essays: instead of using discourse markers, the AI models provide a clear logical structure by separating the different arguments into paragraphs, thereby reducing the need for discourse markers.

Our data also shows that hallucinations are not a problem in the setting of argumentative essay writing: the essay topics are not really about factual correctness, but rather about argumentation and critical reflection on general concepts which seem to be contained within the knowledge of the AI model. The stochastic nature of the language generation is well-suited for this kind of task, as different plausible arguments can be seen as a sampling from all available arguments for a topic. Nevertheless, we need to perform a more systematic study of the argumentative structures in order to better understand the difference in argumentation between human-written and ChatGPT-generated essay content. Moreover, we also cannot rule out that subtle hallucinations may have been overlooked during the ratings. There are also essays with a low rating for the criteria related to factual correctness, indicating that there might be cases where the AI models still have problems, even if they are, on average, better than the students.

One of the issues with evaluations of the recent large-language models is not accounting for the impact of tainted data when benchmarking such models. While it is certainly possible that the essays that were sourced by Stab and Gurevych^41^ from the internet were part of the training data of the GPT models, the proprietary nature of the model training means that we cannot confirm this. However, we note that the generated essays did not resemble the corpus of human essays at all. Moreover, the topics of the essays are general in the sense that any human should be able to reason and write about these topics, just by understanding concepts like ‘cooperation’. Consequently, a taint on these general topics, i.e. the fact that they might be present in the data, is not only possible but is actually expected and unproblematic, as it relates to the capability of the models to learn about concepts, rather than the memorization of specific task solutions.

While we did everything to ensure a sound construct and a high validity of our study, there are still certain issues that may affect our conclusions. Most importantly, neither the writers of the essays, nor their raters, were English native speakers. However, the students purposefully used a forum for English writing frequented by native speakers to ensure the language and content quality of their essays. This indicates that the resulting essays are likely above average for non-native speakers, as they went through at least one round of revisions with the help of native speakers. The teachers were informed that part of the training would be in English to prevent registrations from people without English language skills. Moreover, the self-assessment of the language skills was only weakly correlated with the ratings, indicating that the threat to the soundness of our results is low. While we cannot definitively rule out that our results would not be reproducible with other human raters, the high inter-rater agreement indicates that this is unlikely.

However, our reliance on essays written by non-native speakers affects the external validity and the generalizability of our results. It is certainly possible that native speaking students would perform better in the criteria related to language skills, though it is unclear by how much. However, the language skills were particular strengths of the AI models, meaning that while the difference might be smaller, it is still reasonable to conclude that the AI models would have at least comparable performance to humans, but possibly still better performance, just with a smaller gap. While we cannot rule out a difference for the content-related criteria, we also see no strong argument why native speakers should have better arguments than non-native speakers. Thus, while our results might not fully translate to native speakers, we see no reason why aspects regarding the content should not be similar. Further, our results were obtained based on high-school-level essays. Native and non-native speakers with higher education degrees or experts in fields would likely also achieve a better performance, such that the difference in performance between the AI models and humans would likely also be smaller in such a setting.

We further note that the essay topics may not be an unbiased sample. While Stab and Gurevych^41^ randomly sampled the essays from the writing feedback section of an essay forum, it is unclear whether the essays posted there are representative of the general population of essay topics. Nevertheless, we believe that the threat is fairly low because our results are consistent and do not seem to be influenced by certain topics. Further, we cannot with certainty conclude how our results generalize beyond ChatGPT-3 and ChatGPT-4 to similar models like Bard (https://bard.google.com/?hl=en) Alpaca, and Dolly. Especially the results for linguistic characteristics are hard to predict. However, since—to the best of our knowledge and given the proprietary nature of some of these models—the general approach to how these models work is similar and the trends for essay quality should hold for models with comparable size and training procedures.

Finally, we want to note that the current speed of progress with generative AI is extremely fast and we are studying moving targets: ChatGPT 3.5 and 4 today are already not the same as the models we studied. Due to a lack of transparency regarding the specific incremental changes, we cannot know or predict how this might affect our results.

## Conclusion

Our results provide a strong indication that the fear many teaching professionals have is warranted: the way students do homework and teachers assess it needs to change in a world of generative AI models. For non-native speakers, our results show that when students want to maximize their essay grades, they could easily do so by relying on results from AI models like ChatGPT. The very strong performance of the AI models indicates that this might also be the case for native speakers, though the difference in language skills is probably smaller. However, this is not and cannot be the goal of education. Consequently, educators need to change how they approach homework. Instead of just assigning and grading essays, we need to reflect more on the output of AI tools regarding their reasoning and correctness. AI models need to be seen as an integral part of education, but one which requires careful reflection and training of critical thinking skills.

Furthermore, teachers need to adapt strategies for teaching writing skills: as with the use of calculators, it is necessary to critically reflect with the students on when and how to use those tools. For instance, constructivists^62^ argue that learning is enhanced by the active design and creation of unique artifacts by students themselves. In the present case this means that, in the long term, educational objectives may need to be adjusted. This is analogous to teaching good arithmetic skills to younger students and then allowing and encouraging students to use calculators freely in later stages of education. Similarly, once a sound level of literacy has been achieved, strongly integrating AI models in lesson plans may no longer run counter to reasonable learning goals.

In terms of shedding light on the quality and structure of AI-generated essays, this paper makes an important contribution by offering an independent, large-scale and statistically sound account of essay quality, comparing human-written and AI-generated texts. By comparing different versions of ChatGPT, we also offer a glance into the development of these models over time in terms of their linguistic properties and the quality they exhibit. Our results show that while the language generated by ChatGPT is considered very good by humans, there are also notable structural differences, e.g. in the use of discourse markers. This demonstrates that an in-depth consideration not only of the capabilities of generative AI models is required (i.e. which tasks can they be used for), but also of the language they generate. For example, if we read many AI-generated texts that use fewer discourse markers, it raises the question if and how this would affect our human use of discourse markers. Understanding how AI-generated texts differ from human-written enables us to look for these differences, to reason about their potential impact, and to study and possibly mitigate this impact.

### Supplementary Information

Supplementary Information 1.

Supplementary Information 2.

Supplementary Information 3.

Supplementary Tables.

Supplementary Figures.

## Data Availability

The datasets generated during and/or analysed during the current study are available in the Zenodo repository, 10.5281/zenodo.8343644

## Code availability

All materials are available online in form of a replication package that contains the data and the analysis code, 10.5281/zenodo.8343644.
