# Supplementary material for: A large-scale comparison of human-written versus ChatGPT-generated essays
Source: Sci Rep. 2023 Oct 30;13:18617. doi: 10.1038/s41598-023-45644-9 (PMC10616290; doi:10.1038/s41598-023-45644-9)
Supplement: Supplementary file 1 — Supplementary Information 1. [file 41598_2023_45644_MOESM1_ESM.pdf]

S5 Details about German teacher qualifications

In order to be eligible to teach in German grammar schools, teachers must obtain a state certification. The typical training path for teachers is a five-year university education, during which they study pedagogy and usually two subjects. This is followed by a two-year traineeship at a grammar school, which ends with the state certification examination. After this, teachers at German grammar schools who obtain a position as a civil servant have the rank of *Studienrat* and are evaluated at least every four years. To achieve a higher rank (i.e. *Oberstudienrat* or *Studiendirektor*), teachers apply for promotion on the basis of their current evaluation and seniority. Since the number of higher rank positions is restricted, the achievement of a higher rank depends on (1) the quality of the evaluation, which takes into account aspects of didactic competence, and (2) the length of service according to the Merit Career Act (i.e. *Leistungslaufbahngesetz*). As a result, higher ranks are only achieved after several years of teaching experience.
