# Supplementary material for: A large-scale comparison of human-written versus ChatGPT-generated essays
Source: Sci Rep. 2023 Oct 30;13:18617. doi: 10.1038/s41598-023-45644-9 (PMC10616290; doi:10.1038/s41598-023-45644-9)
Supplement: Supplementary file 2 — Supplementary Information 2. [file 41598_2023_45644_MOESM2_ESM.pdf]

S1 Essay examples from the dataset

Three essays (student essay, ChatGPT-3 generated essay and ChatGPT-4 generated essay) for the topic ‘Should students be taught to compete or to cooperate?’

Student essay: It is always said that competition can effectively promote the development of economy. In order to survive in the competition, companies continue to improve their products and service, and as a result, the whole society prospers. However, when we discuss the issue of competition or cooperation, what we are concerned about is not the whole society, but the development of an individual’s whole life. From this point of view, I firmly believe that we should attach more importance to cooperation during primary education.

First of all, through cooperation, children can learn about interpersonal skills which are significant in the future life of all students. What we acquired from team work is not only how to achieve the same goal with others but more importantly, how to get along with others. During the process of cooperation, children can learn about how to listen to opinions of others, how to communicate with others, how to think comprehensively, and even how to compromise with other team members when conflicts occurred. All of these skills help them to get on well with other people and will benefit them for the whole life.

On the other hand, the significance of competition is that how to become more excellence to gain the victory. Hence it is always said that competition makes the society more effective. However, when we consider about the question that how to win the game, we always find that we need the cooperation. The greater our goal is, the more competition we need. Take Olympic games which is a form of competition for instance, it is hard to imagine how an athlete could win the game without the training of his or her coach, and the help of other professional staffs such as the people who take care of his diet, and those who are in charge of the medical care. The winner is the athlete but the success belongs to the whole team. Therefore without the cooperation, there would be no victory of competition.

Consequently, no matter from the view of individual development or the relationship between competition and cooperation we can receive the same conclusion that a more cooperative attitudes towards life is more profitable in one’s success.

ChatGPT-3 essay: Education is not only about acquiring knowledge, but also about developing the skills and attitudes necessary to succeed in life. One of the most important decisions in education is whether students should be taught to compete or to cooperate.

On the one hand, competition is often seen as a way to motivate students to work harder and achieve more. By pitting students against one another, it is believed that they will be pushed to excel and develop important skills such as resilience, determination, and strategic thinking. In a competitive environment, students may also learn to be independent and self-reliant, which are valuable traits in the real world.

On the other hand, cooperation is equally important in preparing students for their future. By working together, students learn to communicate, share ideas, and build relationships. Cooperation also helps to promote empathy and respect for others, as students learn to appreciate the strengths and weaknesses of their peers. Furthermore, in many industries and careers, collaboration and teamwork are essential for success, and students who are not taught to cooperate may struggle to adapt to these environments.

In conclusion, both competition and cooperation have their advantages and disadvantages, and both are important skills for students to develop. However, it is essential that educators strike a balance between the two, teaching students when to compete and when to cooperate, and providing opportunities to practice both skills. Ultimately, the goal should be to prepare students for the diverse and complex challenges they will face in the future, whether they arise from competition or cooperation.

ChatGPT-4 essay: The contemporary education system frequently grapples with the question of whether students should be taught to compete or cooperate. Striking the right balance is essential, as both competition and cooperation have their merits in shaping a well-rounded individual.

Competition can act as a powerful motivator, pushing students to excel and surpass their perceived limitations. It fosters a spirit of resilience, adaptability, and innovation, as learners strive to outperform their peers. Moreover, competition prepares students for real-world challenges, where they are bound to encounter similar competitive scenarios in their professional lives.

However, solely focusing on competition can lead to excessive stress, anxiety, and unhealthy rivalry, which may hinder students’ personal and academic growth. This is where the value of cooperation becomes apparent. By

teaching students to cooperate, educators encourage essential skills like teamwork, communication, empathy, and problem-solving. These skills are indispensable in today's increasingly interconnected world, where success often depends on the ability to collaborate with others.

In conclusion, the ideal education system should strike a balance between competition and cooperation. While competition nurtures a drive for excellence and adaptability, cooperation fosters critical interpersonal skills and mutual support. By integrating both elements, educators can help shape well-rounded individuals who are prepared to excel in their personal and professional lives.
