# Supplementary material for: A large-scale comparison of human-written versus ChatGPT-generated essays
Source: Sci Rep. 2023 Oct 30;13:18617. doi: 10.1038/s41598-023-45644-9 (PMC10616290; doi:10.1038/s41598-023-45644-9)
Supplement: Supplementary file 3 — Supplementary Information 3. [file 41598_2023_45644_MOESM3_ESM.pdf]

S2 Examples for language complexity

Example 1 is one of the simpler sentences from the student essay from our dataset which can be found in supplemental material S1. This sentence has zero clauses and its maximum dependency tree depth is three. The sentence in Example 2 from the same student essay is significantly longer with four clauses and a maximum dependency tree depth of nine.

1. *The greater our goal is, the more competition we need.*
2. *Take Olympic games which is a form of competition for instance, it is hard to imagine how an athlete could win the game without the training of his or her coach, and the help of other professional staffs such as the people who take care of his diet, and those who are in charge of the medical care.*
