# Supplementary material for: A large-scale comparison of human-written versus ChatGPT-generated essays
Source: Sci Rep. 2023 Oct 30;13:18617. doi: 10.1038/s41598-023-45644-9 (PMC10616290; doi:10.1038/s41598-023-45644-9)
Supplement: Supplementary file 4 — Supplementary Tables. [file 41598_2023_45644_MOESM4_ESM.pdf]

S3 Details about the variables collected within the questionnaire

Supplementary Table 1. Skill levels for self-assessment for reading proficiency, their CEFR equivalents, and the score on the underlying Likert scale.

Self-assessment	CEFR	Score
Comparable to native speaker	C2	5
Business fluent	C1	4
Fluent	B2	3
Good	B1	2
Good basic skills	A2	1
Some basic skills	A1	0

Supplementary Table 2. Scale for the self-assessment of the confidence in the essay ratings.

Self-assessment of rating confidence	Score
I am certain that my assessment is correct.	4
I am fairly certain that my assessment is correct.	3
I am fairly certain that my assessment is correct, but I may have misjudged some aspects.	2
I would defend my assessment, but it is possible that I misjudged aspects.	1
I am not familiar with the topic. My assessment is based on guesses.	0

Supplementary Table 3. Categories and levels for the rating of the essays.

Category	Description of levels from best to worst	Score
Topic and completeness	Addressed the topic to a special degree and considered all aspects of the task.	6
	Addressed the topic in a good manner and considered all aspects of the task.	5
	Addressed the topic and considered most aspects of the task.	4
	Mostly addressed the topic, considered only some aspects of the task.	3
	Topic still discernable, superficial and one-sided, of digresses and repeats itself.	2
	Topic somewhat discernable, every superficial and one-sided.	1
	No relation to the topic.	0
Logic and composition	Clear structure and logical and profound reasoning.	6
	Logical and consistent structure, sound reasoning.	5
	Mostly logical structure, sometimes chains of individual thoughts.	4
	Errors in the logical structure, thoughts not presented coherently.	3
	Logical structure recognizable, jumps in reasoning.	2
	Logical structure recognizable, few thoughts brought to a conclusion.	1
	No logical structure.	0
Expressiveness and comprehensiveness	Very comprehensive and detailed depiction, consistently persuasive.	6
	Comprehensive and detailed depiction.	5
	Partially detailed and mostly comprehensive.	4
	Few details, but still comprehensive.	3
	Somewhat comprehensive.	2
	Almost not comprehensive.	1
	Not comprehensive.	0
Language mastery	Almost perfect language use, few minor rule violations that do not influence the understandability.	6
	Some minor rule violations that do not have a major influence on the understandability.	5
	Some rule violations that affect the understandability.	4
	Multiple rule violations that affect the understandability.	3
	Many rule violations that affect the understandability.	2
	Many severe rule violations that affect the understandability.	1
	Unintelligible language use.	0
Complexity	Often uses complex sentences and sentence links.	6
	Multiple uses complex sentences and sentence links.	5
	Few uses of complex sentences, but multiple simple sentence links.	4
	Consistently simple but correct sentence structures, some sentence links.	3
	Lack of complex language, errors in sentence structures, few sentence links.	2
	Severere errors in sentence structures, no sentence links.	1
	No discernable sentence structures.	0
Vocabulary and text linking	Very comprehensive and variable use of vocabulary and consistent and correct linking of text elements.	6
	Comprehensive and variable use of vocabulary, often correct linking of text elements.	5
	Fundamental vocabulary, sometimes correct linking of text elements.	4
	Limited vocabulary, rudimentary linking of text elements.	3
	Limited vocabulary, sometimes bad choice of words, no linking of text elements.	2
	Strongly limited vocabulary, often bad choice of words, no linking of text elements.	1
	No consistent English vocabulary.	0
Language constructs	Confident use of a large number of language constructs.	6
	Confident use of language constructs.	5
	Mostly correct use of language constructs.	4
	Only few language constructs and partially wrong use.	3
	Predominantly wrong use of language constructs.	2
	Almost always wrong use of language constructs.	1
	Language constructs not following English language rules.	0
