# Supplementary material for: A large-scale comparison of human-written versus ChatGPT-generated essays
Source: Sci Rep. 2023 Oct 30;13:18617. doi: 10.1038/s41598-023-45644-9 (PMC10616290; doi:10.1038/s41598-023-45644-9)
Supplement: Supplementary file 5 — Supplementary Figures. [file 41598_2023_45644_MOESM5_ESM.pdf]

S4 Supporting visualizations for the data analysis

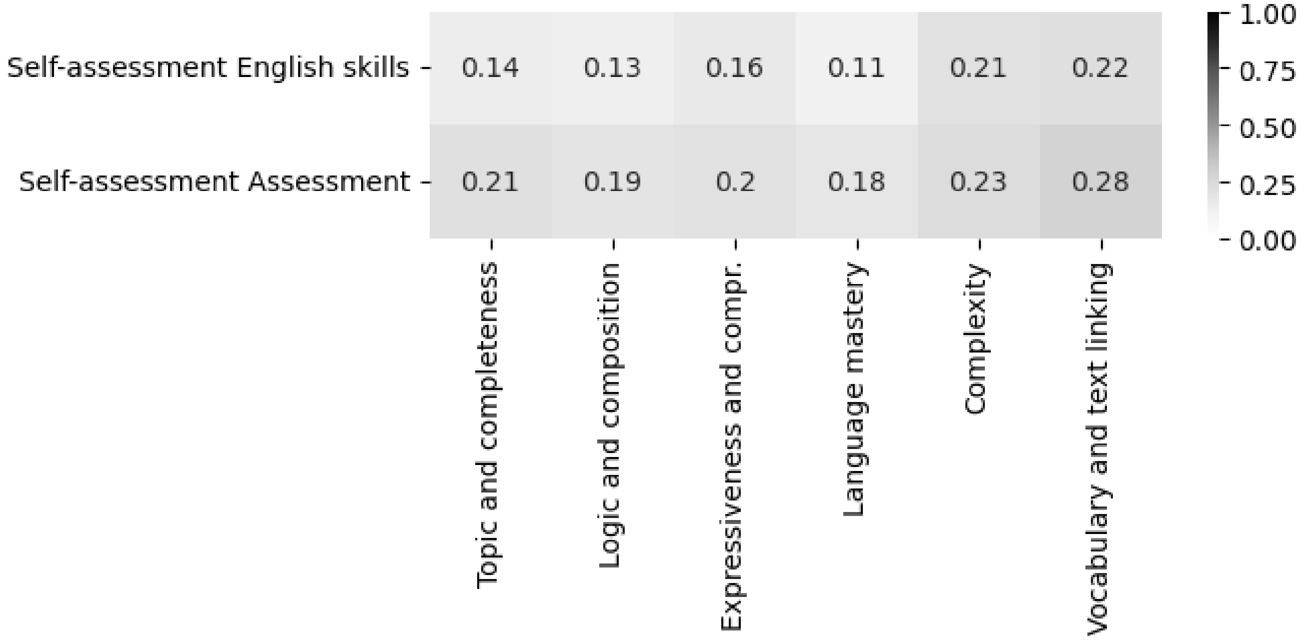

Supplementary Figure 1. Pearson's *r* correlation between the self-assessments. All correlations are significant.

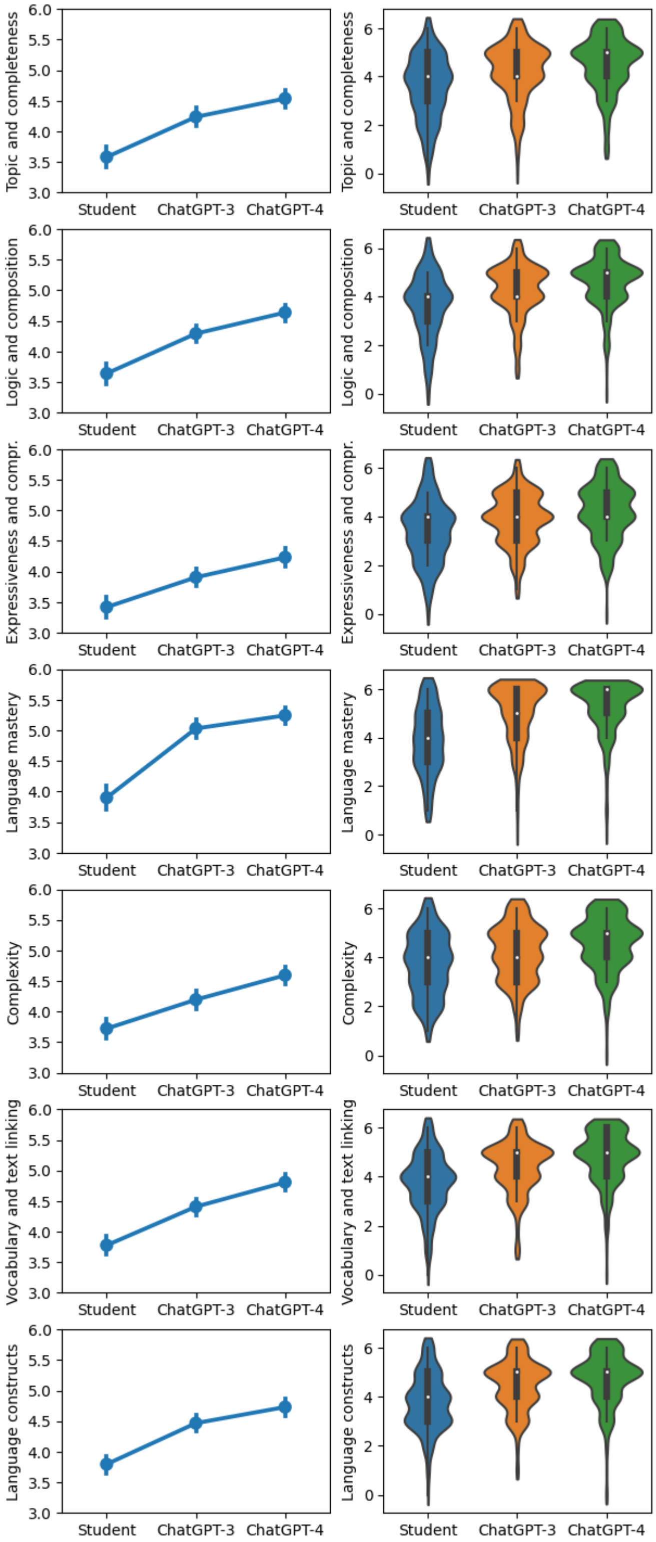

Supplementary Figure 2. Left: Point plots depicting the mean values and confidence intervals of the survey results. Right: Violin plots depicting the distribution of the ratings.

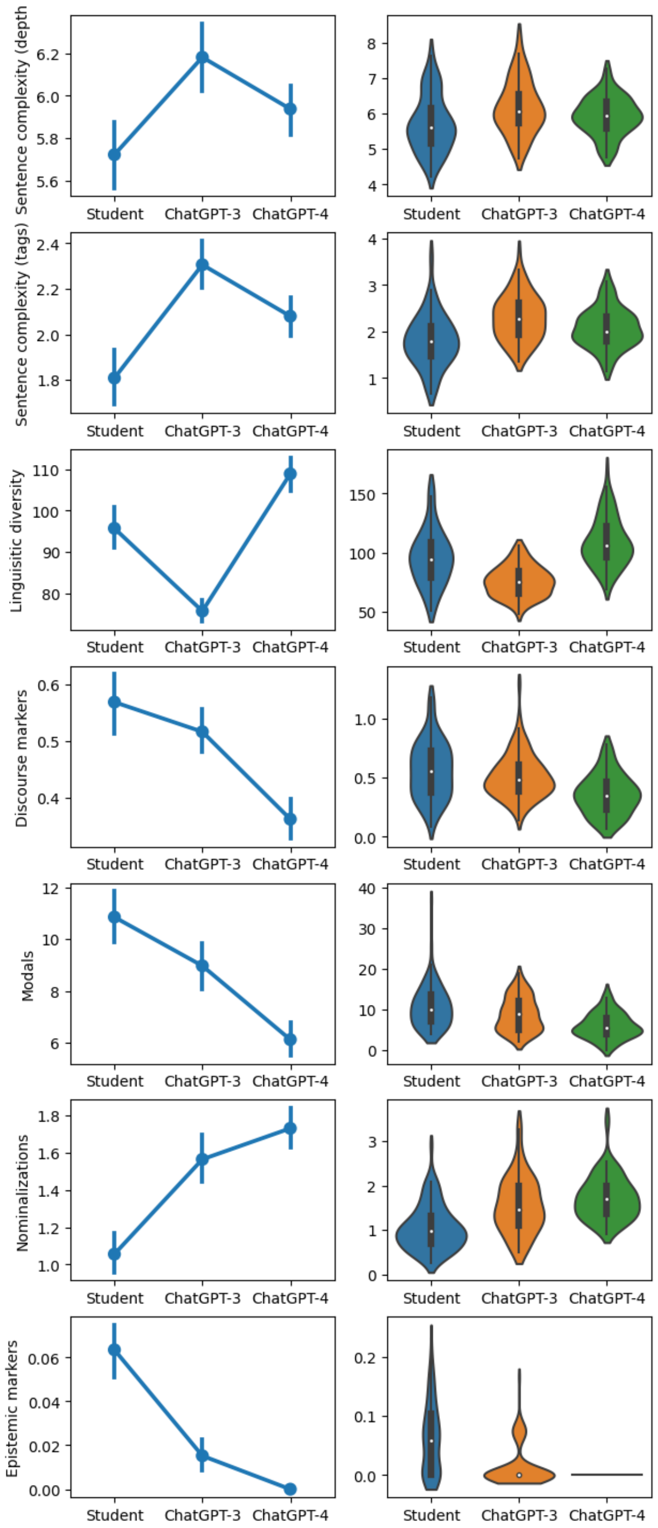

Supplementary Figure 3. Left: Point plots depicting the mean values and confidence intervals of the linguistic characteristics. Right: Violin plots depicting the distribution of the linguistic characteristics.
